# Combined FHL transfer and SpeedBridge reconstruction for calcific avulsion in insertional Achilles tendinopathy: a case report

**DOI:** 10.1097/RC9.0000000000001009

**Published:** 2026-09-21

**Authors:** Julien Paquot, David Roche, Ronny Lopes

**Affiliations:** Centre Orthopedique Santy, FIFA Medical Center of Excellence, Hôpital Privé Jean Mermoz, Groupe Générale de Santé-Ramsay, Lyon, France

**Keywords:** Achilles tendon reconstruction, case report, double-row fixation, flexor hallucis longus tendon transfer, insertional Achilles tendinopathy

## Abstract

**Introduction and importance:**

Insertional Achilles tendinopathy (IAT) is a chronic degenerative condition characterized by pain and structural changes at the calcaneal insertion. Although conservative management remains first-line treatment, a substantial proportion of patients ultimately require surgical intervention. Several surgical strategies have been described for refractory IAT, including debridement, posterior calcaneal osteoplasty, tendon detachment with reinsertion using single- or double-row anchors, and augmentation techniques such as flexor hallucis longus (FHL) tendon transfer. Massive intratendinous calcifications complicated by acute avulsion at the tendon insertion are rare.

**Presentation of case:**

A 52-year-old competitive handball player presented with acute posterior heel pain sustained during sport. Imaging revealed the avulsion of a large insertional calcific deposit associated with Achilles tendon rupture and poor-quality residual fibers. Surgical management consisted of extensive debridement, posterior calcaneal osteotomy, FHL tendon transfer, and double-row SpeedBridge fixation. At 24 months postoperatively, the patient had returned to competitive sport at his pre-injury level with excellent functional outcomes and no complications.

**Clinical discussion:**

In cases of extensive tendon degeneration and calcific avulsion, primary repair alone may be insufficient. Combining biological augmentation with FHL transfer and mechanical reinforcement using double-row fixation may improve structural integrity and functional recovery.

**Conclusion:**

Massive calcific avulsion with substantial tendon loss represents a severe form of insertional Achilles disease. In high-demand patients, combining FHL transfer with double-row fixation may restore tendon continuity and enable return to sport.

## Introduction

Insertional Achilles tendinopathy (IAT) is a chronic degenerative disorder characterized by pain, functional limitation, and structural changes at the tendon’s calcaneal insertion. IAT is frequently associated with intratendinous calcifications, enthesophytes, and degenerative tendinosis[1]. Conservative management, including load modification, physiotherapy, and shockwave therapy, remains the first-line treatment^[^2,3^]^. However, previous studies[4] have reported that 24–45% of patients with IAT ultimately fail nonoperative treatment and require surgical intervention.

HIGHLIGHTS

- Acute calcific avulsion in insertional Achilles tendinopathy is rare.
- Extensive tendon degeneration may preclude primary Achilles repair.
- A flexor hallucis longus tendon transfer provides effective biologic augmentation.
- Double-row fixation improves mechanical stability at the tendon insertion.
- Combined reconstruction enabled a full return to high-level sport.

Several surgical strategies have been described for refractory IAT, including isolated debridement, posterior calcaneal osteoplasty, tendon detachment with reinsertion using single- or double-row anchors, and augmentation techniques such as flexor hallucis longus (FHL) tendon transfer, particularly in cases of tendon substance loss following debridement or excision of calcifications^[^5,6^]^.

Massive intratendinous calcifications in IAT are rare and may occasionally present as an acute fracture-avulsion at the tendon insertion[7]. In such cases, extensive debridement may result in substantial tendon loss, making isolated repair insufficient.

The present case is original in that it required the combination of two techniques – FHL transfer and double-row SpeedBridge (Arthrex, Naples, FL, USA) reconstruction, which are typically used independently.

This case report has been reported in line with the SCARE 2025 guidelines[8].

## Case presentation

A 52-year-old otherwise healthy male competitive handball player (Tegner score 7)[9], measuring 1.90 m and weighing 103 kg, presented in November 2023 with acute onset of left posterior heel pain that occurred during handball practice. His medical history was notable for a prior surgical treatment for right-sided IAT in 2022, with full recovery and return to competitive sport at the same level.

Before the acute injury, the patient reported a history of intermittent insertional pain in the left Achilles tendon, consistent with IAT. Conservative management, including activity modification, physiotherapy, and symptomatic treatment, had provided only partial relief, and symptoms persisted during sports activities.

At presentation, the patient reported a sudden sharp pain followed by immediate functional impairment, sustained during an abrupt change of direction while playing a handball match. Clinical examination revealed localized swelling, marked tenderness at the Achilles tendon insertion, reduced plantarflexion strength, and a palpable defect. Standard radiographs demonstrated avulsion of an insertional calcification (Fig. 1). Magnetic resonance imaging (MRI) confirmed a rupture of the left Achilles tendon at its calcaneal insertion, with poor-quality deep tendon fibers and advanced degenerative changes, indicating that primary tendon repair would be insufficient.

**Figure 1.⋆:**
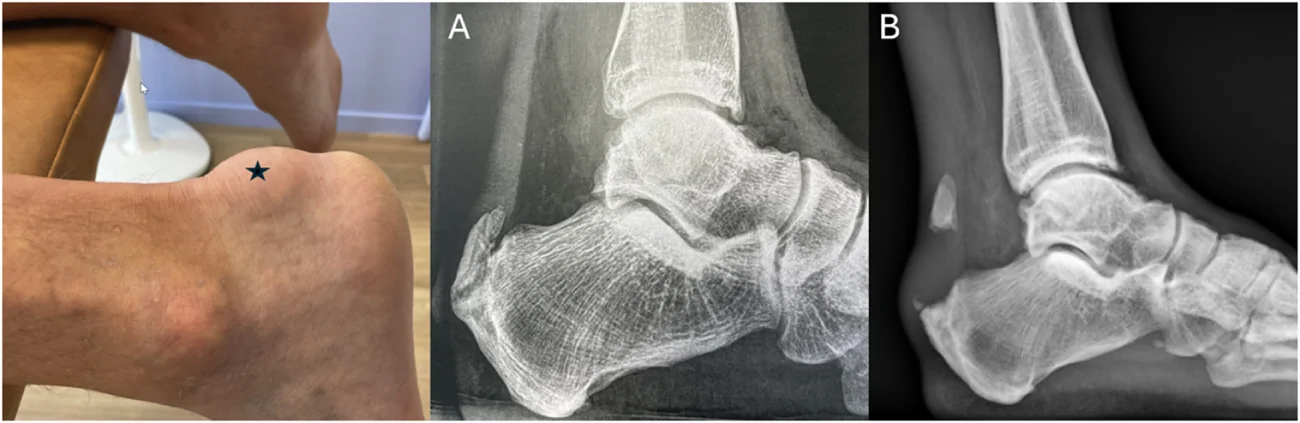
Swelling at the Achilles tendon insertion: (A) radiograph before avulsion and (B) radiograph after avulsion.

Given the extent of tendon degeneration and the presence of a large, displaced calcific fragment, surgical reconstruction was indicated.

Surgery was performed in December 2023 by a senior foot and ankle surgeon with over 15 years of specialized experience. The patient was positioned prone, with a cushion placed under the ipsilateral thigh to maintain the ankle in a neutral position. The foot and ankle were positioned at the edge of the operating table to allow unrestricted ankle motion throughout the procedure. A thigh tourniquet was applied, and prophylactic intravenous antibiotics were administered preoperatively. A regional nerve block combined with general anesthesia was used. Standard skin preparation and sterile draping of the operative limb were performed. A posteromedial approach was used to expose the ruptured Achilles tendon and the fractured calcified enthesophyte. The superficial tendon fibers were detached, while the deep fibers remained partially attached, but exhibited poor structural integrity. Complete excision of the calcific fragment was performed (Fig. 2A and B). A posterior calcaneal osteotomy was then carried out to recreate a healthy tendon insertion footprint. The FHL tendon was harvested through a medial arch incision, prepared using a FiberLoop suture (Arthrex, Naples, FL, USA), and transferred into a 6-mm central calcaneal tunnel. Fixation was achieved using a 6.25-mm interference screw (Fig. 2C–E). Additional reinforcement was performed using a double-row SpeedBridge (Arthrex, Naples, FL, USA) construct with four anchors (Fig. 2F).

**Figure 2.**
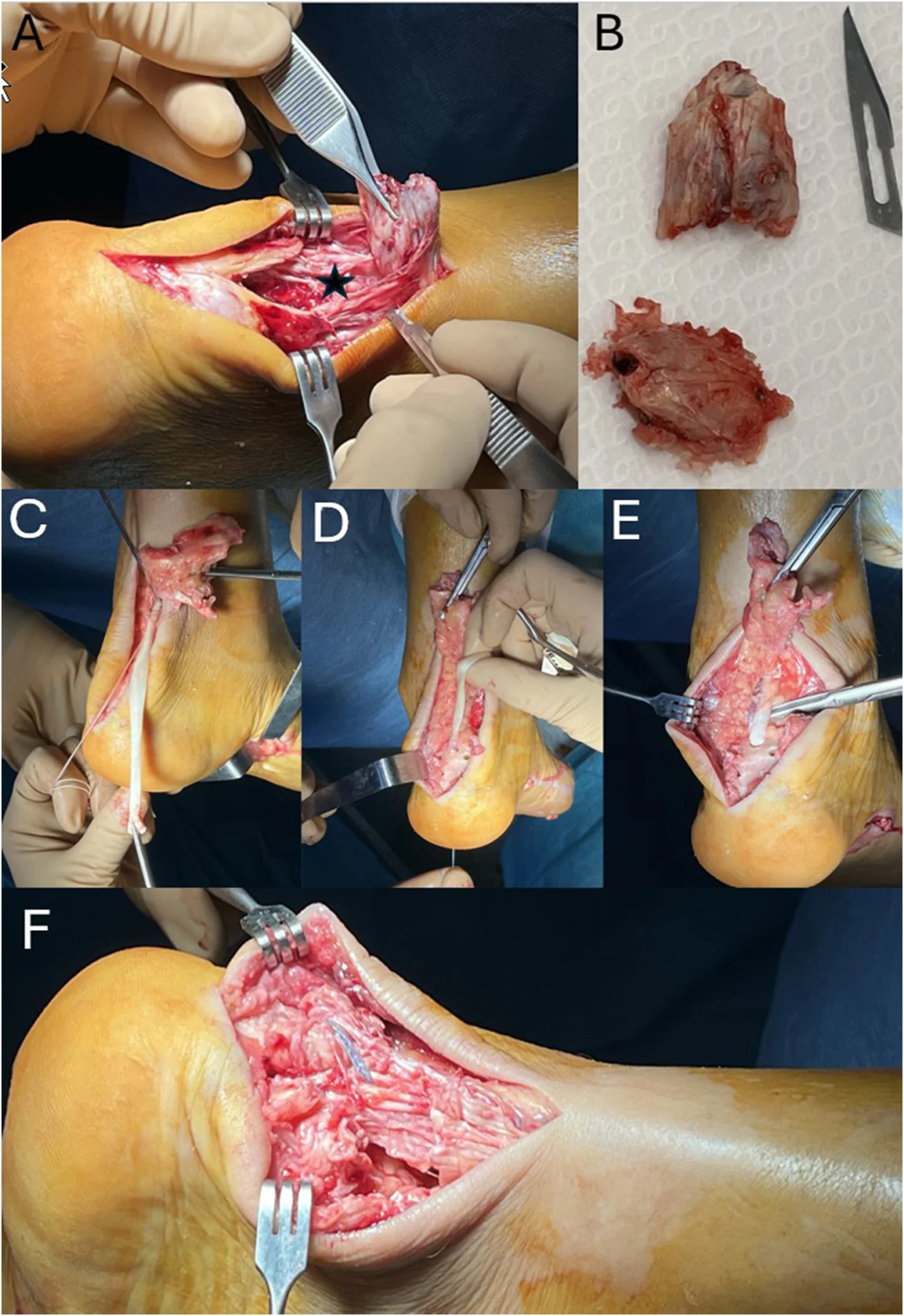
Surgical procedure. (A) Dissection of the calcification and (B) excision of the calcification, revealing poor integrity of the deep tendon fibers: ⋆. (C) Harvesting and preparation of the FHL. (D) Insertion of the FHL into the central calcaneal tunnel. (E) Fixation of the FHL within the tunnel. (F) SpeedBridge augmentation.

Postoperatively, the patient was immobilized in a rigid cast boot in slight plantarflexion, with strict non-weight-bearing maintained for the first 4 weeks. Thromboprophylaxis was prescribed during this period, and level 1 and 2 analgesics were administered as required. At 1 month postoperatively, the boot was removed, and the patient progressed to gradual weight-bearing while initiating a supervised physiotherapy program focused on ankle mobilization, strengthening, and gait retraining. The patient fully adhered to postoperative immobilization and rehabilitation protocols, as assessed during scheduled follow-up visits.

At 4 months postoperatively, the patient walked without a limp and was able to perform both toe and heel raises without pain. At the 1-year follow-up (December 2024), MRI demonstrated satisfactory reinsertion of both the Achilles and FHL tendons without signs of inflammation (Fig. 3). Clinically, the patient had returned to competitive handball at his pre-injury level (Tegner 7).

**Figure 3.**
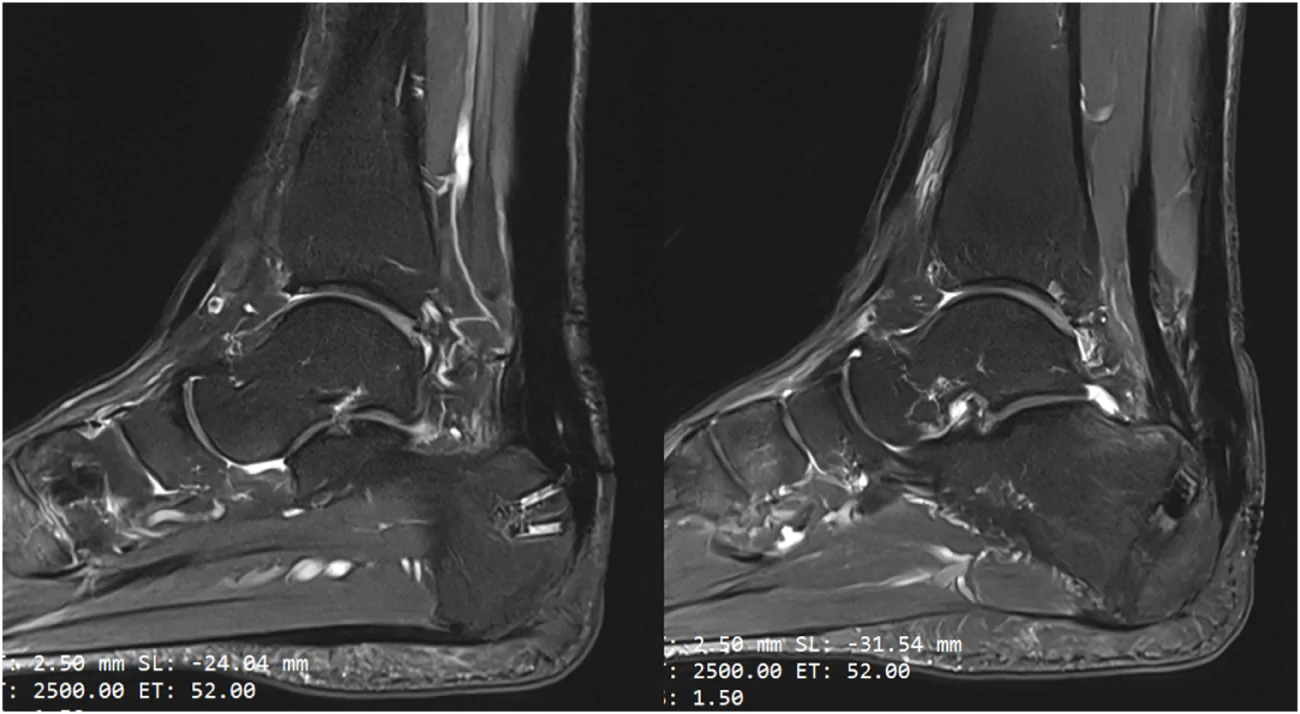
One-year postoperative MRI follow-up demonstrating satisfactory tendon integration.

At the most recent follow-up, 24 months postoperatively in December 2025, the patient remained pain-free with symmetric bilateral function. Bilateral and single-leg heel-rise tests were comparable to those of healthy individuals (Supplemental Digital Content Video 1, available at: https://links.lww.com/IJSCR/A164). He had returned to unrestricted high-demand sports activities, with VISA-A score of 100[10], ALR-RSI score of 100[11], SAV score of 90[12], and EFAS score of 20 and EFAS Sport score of 14[13].

## Discussion

The surgical management of IAT typically includes debridement of the diseased insertion, excision of calcific deposits, posterior calcaneal osteoplasty, and reattachment of the Achilles tendon using anchor fixation. In some cases, some authors also advocate FHL tendon transfer as an adjunctive reconstructive option^[^14,15^]^.

Multiple studies have demonstrated that, in well-selected patients and with meticulous surgical technique, operative treatment of refractory IAT can result in significant functional improvement with a minimum 2-year follow-up^[^16,17^]^.

When extensive tendon substance loss occurs following excision of large intratendinous calcifications or degenerative tissue, augmentation becomes necessary. FHL tendon transfer has been associated with favorable clinical outcomes, with recent evidence showing significant postoperative improvement in function, pain relief, and high patient satisfaction[18]. These findings are consistent with longer-term series reporting sustained functional recovery after FHL tendon transfer in chronic Achilles tendon pathology, with mean follow-up durations of approximately 4 years[19].

Double-row anchor fixation has also gained attention because of its biomechanical advantages. Compared with single-row repairs, double-row constructs provide a larger tendon-bone footprint, greater stability[20], and improved load distribution[21].

In the present case, alternative surgical strategies such as primary reattachment, V-Y tendon advancement, or augmentation using tendon grafts were considered but deemed inappropriate^[^19,22^]^. The severity of tendon degeneration and the size of the calcific avulsion left insufficient residual tendon for a reliable primary repair. Moreover, V-Y advancement or graft augmentation may lengthen the musculotendinous unit and compromise plantarflexion strength, an unacceptable drawback for a high-demand athlete.

The potential benefit of FHL transfer depends on preserving the mechanical contribution of the native Achilles insertion. Recent biomechanical data suggest that performing an isolated FHL transfer without reattaching the Achilles stump results in a significant reduction in ankle torque at low angular velocity[18]. Therefore, restoring continuity of the Achilles insertion whenever possible remains critical to optimize functional outcomes.

In addition, double-row anchor fixation provides inherent biomechanical advantages by increasing the tendon–bone contact area, improving load distribution and initial construct stability^[^20,21^]^. These considerations supported our choice to combine FHL transfer with a robust double-row SpeedBridge (Arthrex, Naples, FL, USA) reconstruction to optimize both biological augmentation and mechanical strength.

Massive calcific avulsion at the Achilles insertion represents a severe manifestation of degenerative insertional disease. When extensive debridement compromises tendon integrity, combining biological augmentation with FHL transfer and a structurally robust double-row construct may help restore tendon continuity and plantarflexion strength. In the present case, this strategy resulted in excellent functional recovery and a return to sport. However, given the nature of a single case report, further studies are required to better define its indications and reproducibility.

### Strengths and limitations

This case highlights a rare and severe form of insertional Achilles pathology that was managed with a combined reconstructive approach, with a documented 24-month functional recovery. However, because this is a single case, the results cannot be generalized and require validation in larger series.

## Conclusion

This case report describes a rare presentation of insertional Achilles pathology with an acute avulsion of a massive calcific deposit in a 52-year-old active athlete.

Surgical management, combining extensive debridement, FHL tendon transfer, and double-row fixation using a SpeedBridge construct, resulted in the restoration of tendon continuity, excellent functional recovery, and return to high-level sport.

Although conclusions regarding superiority cannot be drawn from a single case report, this reconstructive strategy may be considered when substantial tendon loss precludes reliable primary repair. Further studies are needed to determine its reproducibility and long-term outcomes.

## Data Availability

Data sharing is not applicable to this article as no datasets were generated or analyzed beyond those included in the case description.

## Patient perspective

The patient reported high satisfaction with the surgical outcome and rehabilitation process. He expressed confidence in the operated ankle and described a successful return to competitive handball without pain or functional limitation.
